# Does clinical governance influence the appropriateness of hospital stay?

**DOI:** 10.1186/s12913-015-0795-2

**Published:** 2015-04-03

**Authors:** Maria Lucia Specchia, Andrea Poscia, Massimo Volpe, Paolo Parente, Silvio Capizzi, Andrea Cambieri, Gianfranco Damiani, Walter Ricciardi, OPTIGOV© Collaborating Group, Antonio Giulio De Belvis

**Affiliations:** Department of Public Health, Catholic University of Sacred Hearth, Largo F.Vito, 1, 00168 Rome, Italy; Clinical Directorate “A. Gemelli” Teaching Hospital, Largo Gemelli 8, 00168 Rome, Italy

**Keywords:** Clinical governance, Quality, Appropriateness, OPTIGOV^©^, Appropriateness Evaluation Protocol

## Abstract

**Background:**

Clinical Governance provides a framework for assessing and improving clinical quality through a single coherent program. Organizational appropriateness is aimed at achieving the best health outcomes and the most appropriate use of resources. The goal of the present study is to verify the likely relationship between Clinical Governance and appropriateness of hospital stay.

**Methods:**

A cross-sectional study was conducted in 2012 in an Italian Teaching Hospital. The OPTIGOV^©^ (*Optimizing Health Care Governance*) methodology was used to quantify the level of implementation of Clinical Governance globally and in its main dimensions. Organizational appropriateness was measured retrospectively using the Italian version of the Appropriateness Evaluation Protocol to analyze a random sample of medical records for each clinical unit.

Pearson-correlation and multiple linear regression were used to test the relationship between the percentage of inappropriate days of hospital stay and the Clinical Governance implementation levels.

**Results:**

47 Units were assessed. The percentage of inappropriate days of hospital stay showed an inverse correlation with almost all the main Clinical Governance dimensions. Adjusted multiple regression analysis resulted in a significant association between the percentage of inappropriate days and the overall Clinical Governance score (β = −0.28; p < 0.001; R-squared = 0.8). EBM and Clinical Audit represented the Clinical Governance dimensions which had the strongest association with organizational appropriateness.

**Conclusions:**

This study suggests that the evaluation of both Clinical Governance and organizational appropriateness through standardized and repeatable tools, such as OPTIGOV^©^ and AEP, is a key strategy for healthcare quality. The relationship between the two underlines the central role of Clinical Governance, and especially of EBM and Clinical Audit, in determining a rational improvement of appropriateness levels.

## Background

The need to improve health care quality while pursuing costs’ rationalization and reduction is currently a common topic in the discussion about health systems efficacy and effectiveness.

Clinical Governance (CG) is a key drive towards quality improvement in health care [1] and enhancement of the corporate responsibility for the quality of care [2]. In fact, CG provides a framework for bringing together all local activities for improving and assessing clinical quality into a single coherent program which encourages everyone in the organization to be a part of and work to improve quality and safety of patient care [3].

With the aim of assessing and measuring the implementation level of CG within health care organizations, in 2006 the Department of Public Health of the Catholic University of the Sacred Heart developed “OPTIGOV^©^” (Optimizing Health Care Governance), a methodology consisting of a hospital audit based on a structured and systematic approach and on an improvement operational plan. OPTIGOV^©^ allows to: analyze the CG structural and functional prerequisites and areas and assign to each of them a score; identify the organization strengths and weaknesses; provide suggestions and indications to be implemented by the Hospital management and monitor the health care services quality improvement [4].

The application of OPTIGOV^©^ within several Italian hospitals resulted in a realistic representation of the effective CG implementation both at the Board level (top management) and the Unit level (each single ward) [5]. Furthermore, the concept of appropriateness in healthcare has progressively become one of the guiding principles of health systems with more and more attention being given to both quality improvement and effectiveness of health care [6].

The continuous evaluation of the efficient hospital utilization is an essential issue that must be considered to increase the quality of health care providers. In fact, unjustified hospital admissions and stays not only increase costs, but are also related to poor health services [7].

The Appropriateness Evaluation Protocol (AEP) is a widely used assessment tool that identifies and measures the inappropriateness variables of hospital healthcare related to unjustified admission and/or length of stay [8]. Moreover, the AEP has been designed to determine the reasons of inappropriate use of hospitals with the aim of strengthening health care professionals’ awareness and supporting decision-making [9].

Several studies have shown that the application of CG tools, as Clinical Audit or Health Technology Assessment, could positively impact on organizational appropriateness in hospital settings [9,10]. However, despite the progressive increase of health professionals’ interest in appropriateness and CG themes [11], no practical study about their connection is available.

The purpose of this study was to verify the likely relationship between the appropriateness of hospital stay and the CG implementation level, by comparing the results of OPTIGOV^©^ methodology and AEP applications within a large Teaching Hospital.

## Methods

The study was conducted between July and December 2012 in an Italian Teaching Hospital with the aim to simultaneously represent CG implementation levels, as measured through OPTIGOV^©^, and organizational appropriateness, as measured through AEP.

### OPTIGOV^©^

The OPTIGOV^©^ methodology is aimed at assessing the CG implementation level within a health care organization by assigning an overall CG score and partial scores referred to the single CG areas (min = 0 – max = 100). OPTIGOV^©^ constitutive elements, characteristics and steps have been previously described in detail [4].

A joint project team from the Department of Public Health and the Hospital Top Management, consisting of 4 public health experts and 2 health economists, assessed the CG implementation at the Unit level (47 clinical wards) in each of the 10 Departments of the Teaching Hospital.

The CG areas analyzed were represented by: Evidence Based Medicine (EBM), Accountability, Clinical Audit, Risk Management, Performance Evaluation, Patient Involvement. These areas were assessed through hospital audits, supported by an assessment tool: the OPTIGOV^©^ Scorecard.

The EBM area was assessed as the practice of medicine based on the integration of the physician’s clinical experience with the best scientific proof available applied to each patient’s unique features and values.

The Accountability area was tested as the availability within the organization of univocal systems of identification of those responsible for the clinical procedures (doctors, nurses and other health professionals).

The Clinical Audit area was estimated as the organizational level and the quality of organized and structured peer reviews, aimed at systematically examining one’s own activity and results by comparing these with explicit standards, with the purpose of improving healthcare quality and outcomes.

The Performance Evaluation area was assessed by evaluating the ability of the healthcare organization and units to systematically monitor the results of clinical practice in terms of efficacy, suitability, efficiency, quality and time.

The techniques and methods to manage risk, the existence of insurance coverage, the identification of risks, the procedures to prevent risks and medical errors have been evaluated in the “Risk Management” area.

Finally, for the “Patient Involvement” area the structured and systematical methods of discussion and dialogue with the patient/citizen about clinical decisions taken in healthcare wards were assessed.

A CG global score and 6 partial scores referred to the above mentioned CG areas were obtained by applying the OPTIGOV^©^ Scorecard [4].

At the end of the OPTIGOV^©^ evaluation a feedback to the Heads of the Departments and of the Units was given through specific written reports. Moreover several meetings with health professionals were organized to display and discuss the overall results.

### AEP

The AEP evaluation is part of a hospital program started in 2012 to improve appropriateness levels. The methodology used for the AEP evaluation is described in detail in a methodological paper [12] and briefly summarized below.

An evaluation team, consisting of one medical doctor and one nurse, carried out independently a retrospective assessment of the organizational appropriateness of the Teaching Hospital. The assessment was performed by analyzing a random sample of medical records through the application of the Italian version of the AEP [13]. Both the physician and the nurse were trained by a physician expert in Public Health with certified know-how in the use of PRUO in Italy [14,15]. However, the physician was already familiar with AEP given his collaboration with the Italian research group on the obstetric PRUO [16]. The PRUO manual was used for training and for reference during reviews. The reviewers only used the criteria included in the PRUO manual. The override criteria were not used (neither positive, nor negative). Each day of hospital stay was considered appropriate if it met one of the AEP criteria. Both the physician and the nurse reviewed all the records without the knowledge of the other’s decision. In case of different judgment, a final agreement about appropriateness was reached by the two evaluators through the discussion and comparison of the different points of view.

1371 records from 47 Units, that represented about 3% of the whole hospital activity as suggested for the regional standard about appropriateness evaluation [17], was sampled. Organizational appropriateness of hospital stay was measured as the frequency of inappropriate days on the whole days of hospital stay.

At the end of the AEP evaluation, the hospital management gave a feedback to the Heads of Departments/ Units through specific written reports and several meetings with health professionals were organized during which the results of the assessment were shown and discussed.

### Statistical analysis

A bivariate analysis was carried out between the outcome variable (frequency of inappropriate days) and each variable included in OPTIGOV^©^ and AEP surveys. Pearson pairwise correlation was used: if the p value was lower than 0.25, the variable was tested for association with a multiple linear regression model which was used to test the likely influence of the selected variables on organizational appropriateness of hospital stay. Since type of admission (elective/urgent), type of hospital ward (surgical/medical) and appropriateness in admissions (as measured by AEP) are likely to influence hospital appropriateness [18,19], these were included as covariates in the regression analysis. The regression equation was:

$$ {\mathrm{Y}}_{\mathrm{i}} = {\upbeta}_0 + {\upbeta}_1{\mathrm{X}}_1 + {\upbeta}_2{\mathrm{X}}_2 + {\upbeta}_3{\mathrm{X}}_3 + {\upbeta}_4{\mathrm{X}}_4 $$

Where Y_i_ = organizational appropriateness of hospital stay; β_0_ = constant; β_1–4_ = weights of the variables; X_1_ = selected variable dealing with OPTIGOV^©^ (CG overall score in Table 1, EMB score in Table 2 and Clinical audit score in Table 3); *X*_2_ = percentage of urgency admissions; X_3_ = type of hospital ward; X_4_ = appropriateness in admissions.

**Table 1:** **CG global and partial scores**

	**Mean**	**SD**	**Min**	**Max**	**95% CI**
Clinical Governance	48.84	14.25	27.99	85.59	43.63 - 51.84
EBM	49.00	13.21	15.48	85.71	44.62 - 52.24
Accountability	59.03	19.37	28.57	100.00	53.03 - 63.87
Clinical Audit	36.50	29.41	3.77	97.80	25.96 - 42.38
Performance Evaluation	60.00	18.67	18.75	100.00	53.47 - 63.89
Risk Management	26.53	26.08	00.00	100.00	18.12 - 32.69
Patient Involvement	51.97	14.57	29.41	94.12	57.11 - 65.50

SD: standard deviation; CI: confidence interval.

**Table 2:** **Results of the AEP application**

	**Mean**	**SD**	**Min**	**Max**	**95% CI**
Medical Records	29.17	20.43	8	125	
Days evaluated (AEP)	143.53	83.80	32	400	
% inappropriateness (days)	27.69%	19.21%	0.00%	70.90%	22.04% -33.33%
% appropriateness in admissions	63.01%	24.86%	20.00%	100.00%	55.71% - 70.31%
% admissions in urgency*	37.78%	27.80%	0.01%	98.9%	29.42% -46.12%

SD: standard deviation; CI: confidence interval.

*The percentage of urgent admissions is referred to 45 wards over 47 because of the lack of this information for two Units.

**Table 3:** Overall CG score and inappropriateness of hospital stay (days) *

	**β coefficient**	**95% CI**		***p***
**overall CG score**	−0.28	−0.48	−0.07	<0.01
% admissions in urgency	0.01	−0.11	0.11	0.99
ward (surgical/medical)	0.40	−5.11	5.91	0.88
% appropriateness in admissions	−0.68	−0.79	−0.57	<0.01
constant	83.58	71.16	96.01	<0.01

*The multiple regression included 45 wards over 47 because of the lack of information about the percentage of urgent admissions of two Units.

A *p-*value less than 0.05 was considered statistically significant. Statistical analysis was performed by using STATA 12 software.

### Ethics statement

Approval of the ethics committee was not required for the study because the Italian legislation concerns only clinical research studies and does not provide statements on observational studies on aggregated data collected from administrative databases without the patient’s involvement. For the present study anonymous data about AEP were extracted from routinely collected administrative databases and there was no need to obtain additional data from individual patients. Indeed, in 2012 the Management of the Teaching Hospital involved in this project decided to launch a quality improvement program. The assessment of appropriateness with AEP was carried out by the Medical Direction as a part of this improvement program. For the present study, researchers had access only to an anonymous dataset containing aggregated data (Unit level), which ensured patients’ privacy. For these reasons, no personal informed consent to the present analysis was requested from study participants.

## Results

CG and organizational appropriateness were evaluated in 47 hospital wards (24 surgical and 23 medical wards). The results of OPTIGOV^©^ and AEP applications are shown in Tables 1 and 2.

By means of a multiple regression analysis, we found a statistically significant association between the overall CG score and the percentage of inappropriate hospital stay (days) (β = −0.28; p < 0.001; R-squared = 0.82) (Table 3). Our model suggests that for each unitary increase of the overall CG score, a mean reduction of about 0.3 inappropriate days resulted. Furthermore, the most important variable predicting the inappropriateness rate was the percentage of appropriate admissions, as for a unitary increase in appropriate admission, a mean reduction of about 0.7 inappropriate days resulted, too. Medical wards seemed to be related to a decrease of hospital stay appropriateness, but this correlation did not approach statistical significance (Table 3).

The percentage of inappropriate days of hospital stay was inversely correlated with all the CG dimensions, except for Risk Management (rho = 0.12, p = 0.38). However, among the CG dimensions inversely correlated with inappropriateness, a significant correlation was only found with respect to EBM (rho = 0.34, p < 0.05), while a trend was found with respect to Clinical Audit (rho = 0.27, p = 0.06).

By applying the multiple regression analysis, a significant association between the percentage of inappropriate days and the EBM score (β = −0.33; p < 0.01; R-squared = 0.83) and the Clinical Audit score (β = −0.11; p = 0.02; R-squared = 0.81), was found (Table 4 and 5). Such association was stronger for EBM, as for a unitary increase of the EBM score, a mean reduction of about 0.3 inappropriate days resulted, while for a unitary increase of the Clinical Audit score, the mean reduction was about 0.1.

**Table 4:** EBM score and inappropriateness of hospital stay (days) *

	**β coefficient**	**95% CI**		**p**
**EBM score**	−0.33	−0.53	−0.12	<0.01
% admissions in urgency	0.02	−0.07	0.10	0.95
ward (surgical/medical)	0.64	−4.71	5.99	0.81
% appropriateness in admissions	−0.66	−0.77	−0.55	<0.01
constant	85.27	73.33	97.20	<0.01

*The multiple regression included 45 wards over 47 because of the lack of information about the percentage of urgent admissions of two Units.

**Table 5:** Clinical Audit score and inappropriateness of hospital stay (days) *

	**β coefficient**	**95% CI**		***p***
**Clinical Audit score**	−0.11	−0.21	−0.01	0.02
% admissions in urgency	0.02	−0.09	0.13	0.70
ward (surgical/medical)	0.80	−4.94	6.54	0.78
% appropriateness in admissions	−0.67	−0.79	−0.55	<0.01
constant	72.57	64.69	80.45	<0.01

*The multiple regression included 45 wards over 47 because of the lack of information about the percentage of urgent admissions of two Units.

## Discussion

Our study is the first aiming to explore and detect the relationship between the appropriateness of hospitalization and the CG implementation level. Our results showed an association between CG implementation and appropriateness in hospital care. CG scores, as measured with OPTIGOV^©^, in particular EBM and Clinical Audit, were found to be inversely correlated with appropriateness, as measured with the AEP protocol.

Since CG is a system through which health care organizations are “accountable for continuously improving the quality of their services and safeguarding high standards of care by creating an environment in which excellence in clinical care will flourish” [1], each intervention aimed at improving CG is likely to positively influence healthcare quality and consequently appropriateness of care. In fact, healthcare quality – that is pursued by CG - consists of several measurable dimensions (safety, accessibility, acceptability, appropriateness, provider competence, efficiency, effectiveness and outcomes), one among which is appropriateness of care [20,21]. EBM and Clinical Audit represented the Clinical Governance dimensions mostly associated with organizational appropriateness.

Our main findings can be interpreted as follows. When health care professionals’ activity is based on the integration of their own clinical experience with the best scientific proof available applied to each patient (EBM) and when an organized and structured peer review process (Clinical Audit) is carried out in a rigorous and systematic way, hospital care is more likely to comply with the criteria underlying the concept of appropriateness. Strengthening scientific knowledge and attitude towards its continuous improvement makes clinicians more prone to systematically reflect on the way they supply care, apply protocols, guidelines and, consequently, to reach a better level of appropriateness.

In our Teaching Hospital, the overall CG score, measured through the application of the OPTIGOV^©^ methodology, was 48.8, thus meaning that about half of the theoretical highest score was satisfied in our survey. These CG values are comparable with the results of other experiences carried out within several Italian Hospitals by using the same methodology [5], although CG is widely implemented across the Italian healthcare system [22].

By measuring the level of hospital stay appropriateness through PRUO, we found a mean rate of inappropriateness of 27.6%, ranging from 0.0% to 70.1%. Such inappropriateness rate is similar to that reported by previous studies, even though other papers showed lower (14%) or higher (40%) rates [23,24]. Some studies reported a large heterogeneity in the inappropriateness rates among different hospitals of the same Italian Region, with values ranging from 17.9% to 57.9% [25], while others demonstrated different inappropriateness levels even in the same hospital in different time periods [26].

We adjusted hospital stay appropriateness for appropriateness in admission, type of admission (urgent vs elective) and type of ward (surgical vs medical): a recently published study [27] confirms the importance of inappropriateness in admission which accounts for 14% of acute hospitalizations. In our study such percentage was 27%, and, by applying the regression model, it represented the first predictor of inappropriateness rate.

Nevertheless, our study shows some limits. The first one concerns the psychometric properties of OPTIGOV. Indeed, although the results of its application within several Italian Hospitals lead to hypothesize the validity and reliability of the methodology, carrying out of further studies would be advisable in order to confirm these properties. However, the previous applications of OPTIGOV showed its potential to produce a realistic representation of the CG implementation level of a hospital, highlighting both criticisms and transferable best practices and providing concrete plans for organizational change and quality improvement [4,5]. On the other hand, the validity and reliability of the AEP have been tested extensively. Indeed, in their first work, Gertman and Restuccia found an overall agreement rate between pairs of reviewers from 92 to 94% (73 to 79% if the case was judged inappropriate by at least one of the reviewers) [8]. Moreover, as reported by McDonagh MS et al., reliability testing shows a specific agreement rate of 24–75% for admissions and 64–85% for days of hospital stay [28]. These ranges have been confirmed by Lorenzo et al. for the European version of the AEP [29]. Finally, validity specific agreement rate has been reported to range from 39 to 80% for admissions and 59–91% for days of hospital stay [28]. Unfortunately, we did not test the inter-rater reliability in our study.

Furthermore, both OPTIGOV and AEP are based on the detection of process indicators which can be considered a proxy measure of overall quality in healthcare. Anyway, other studies already showed that improvements in CG can drive the organization to better performance [30]. Moreover, we have to point out that the study design is cross-sectional. Therefore the huge variability among hospital wards both for CG (27-85%) and appropriateness rates (0-70%), confirmed by the literature [19,23-26], may be due both to health professionals’ attitudes and behaviors, as well to organizational barriers. Such hypothesis can be confirmed through appropriate longitudinal studies.

In addition, further studies would be useful to investigate the effectiveness of specific CG tools and processes in improving appropriateness in hospital care, particularly referring not only to EBM and Clinical Audit, but also to the application of targeted training programs for healthcare professionals and the development of clinical pathways.

In this sense, considering that monitoring represents one of the pillars of continuous healthcare quality improvement, our study has been aimed at investigating the impact of the application of an integrated approach to the evaluation of hospital performance consisting of the combined implementation of rigorous tools. A systematic implementation of such an approach within healthcare organizations would be desirable in the future. It could also play an important role in the definition of hospital financial strategies and incentive systems for healthcare professionals based on pay for performance.

## Conclusion

Improving healthcare efficiency needs awareness and control of unnecessary and inappropriate hospitalization. Both healthcare quality and appropriateness should be pursued through a systemic approach based on CG principles, tools and processes. This study suggests that CG plays a central role in determining a rational improvement of appropriateness levels and recommends the systematic, structured and integrated implementation of standardized and repeatable tools to monitor healthcare quality improvement.

## Abbreviations

OPTIGOV^©^: (*OptimizingHealthCareGovernance*)
CG: Clinical Governance
AEP: Appropriateness Evaluation Protocol
SD: Standard Deviation
CI: Confidence Interval
